# Investigating pediatric nurses’ perceptions of factors contributing to MAEs at Yendi hospital, Ghana

**DOI:** 10.1186/s12887-024-05269-x

**Published:** 2024-12-03

**Authors:** Ruth Nimota Nukpezah, Nathaniel Awenlesakba Anyaba, Wahab Osman

**Affiliations:** 1 School of Nursing and Midwifery, University for Development Studies, Tamale, Ghana; 2 Ghana College of Nurses and Midwives, Accra, Ghana

**Keywords:** Medication administration errors, Pediatric nurses, Patient safety, Ghana, Healthcare settings

## Abstract

**Background:**

Medication administration errors (MAEs) are a critical concern in pediatric healthcare, contributing to adverse drug events (ADEs) and negatively impacting patient health.

**Objectives:**

This study explores pediatric nurses’ perceptions of factors contributing to MAEs at Yendi Municipal Hospital to develop interventions enhancing patient safety.

**Methods:**

A descriptive cross-sectional survey was conducted among 143 nurses at Yendi Municipal Hospital using structured questionnaires. Data were analysed using SPSS 26.0 and Excel 2016. Bivariate analysis examined relationships between socio-demographic characteristics and MAEs.

**Results:**

Contributing factors to MAEs included inadequate training (91.6%), misunderstanding medical abbreviations (88.8%), poor supervision (92.3%), eagerness to sign out shifts (70.6%), improper handover (88.8%), inadequate staff (77.6%), dosage miscalculations (83.9%), and illegible handwriting (81.8%). Significant associations were found between MAEs and the type of unit/ward (X²=6.25, *p* = 0.012) and educational level (Fisher Exact test = 4.20, *p* = 0.036).

**Conclusion:**

Inadequate training, poor supervision, and communication issues are major contributors to MAEs in pediatric settings. Targeted interventions can significantly improve patient safety and care quality.

**Supplementary Information:**

The online version contains supplementary material available at 10.1186/s12887-024-05269-x.

## Introduction

Medication administration errors (MAEs) are recognised globally as a significant and preventable issue in healthcare delivery, particularly in pediatric settings, where the consequences of such errors are often more severe than in adults. Pediatric patients have unique physiological characteristics, including smaller body sizes, developing organ systems, and needing weight-based dosing [1, 2]. As a result, the margin for error is considerably narrower when administering medications to children [3, 4]. Even slight miscalculations in dosage or timing can lead to adverse drug events (ADEs), which can significantly impact a child’s health, potentially leading to prolonged hospitalisation, complications, or, in some cases, mortality (1, 2, 3–4). Recent studies have emphasised that pediatric patients are at an elevated risk for adverse drug events (ADEs) due to these characteristics, with estimates suggesting that the risk of medication errors in children is up to three times higher than in adults [5, 6]. These vulnerabilities are particularly concerning in low- and middle-income countries (LMICs), where the healthcare system infrastructure may not adequately support pediatric-specific care protocols [7]. This underlines the urgency of addressing pediatric-specific medication safety challenges in high- and low-resource settings [8, 9].

Globally, the rate of MAEs in pediatric care is notably higher than in adult care. This is largely due to the complexities involved in pediatric medication, such as the need for individualised dosing based on the child’s weight and developmental stage, as well as the frequent use of adult-formulated drugs that require modification before they can be safely administered to children [10]. Inaccuracies at any stage of the medication process—prescribing, transcribing, dispensing, or administration [11], can have serious consequences for pediatric patients. For instance, incorrect calculations for weight-based dosing or errors in adjusting adult medications for pediatric use are common in pediatric settings and have been linked to an increased risk of ADEs [12, 13].

Despite the urgency of addressing MAEs in pediatric care, most studies and interventions have focused on adult populations, and this leaves a critical gap in understanding the factors contributing to MAEs in pediatric patients, especially in resource-constrained settings such as those in Sub-Saharan Africa [14]. In countries like Ghana, healthcare systems face numerous challenges that exacerbate the risk of medication errors in pediatric settings. These include limited access to pediatric-specific training, inadequate staffing, poor access to essential medications, and insufficient infrastructure to support safe medication administration [3–4, 4, 15]. These systemic barriers often force healthcare workers, particularly nurses, to rely on general or adult-dosing protocols [16, 17], which increases the risk of incorrect medication administration in pediatric patients.

Moreover, Ghana’s healthcare system is characterised by a high patient-to-nurse ratio, particularly in rural settings, where healthcare resources are even more scarce. Nurses, often the primary caregivers responsible for administering medications, frequently face overwhelming workloads and inadequate support [18, 19]. Studies conducted in major teaching hospitals in Ghana have shown that over 60% of nurses reported experiencing high levels of stress and fatigue, both of which are known contributors to medication errors [7, 20]. However, limited research examines how these factors affect pediatric care, particularly in rural healthcare settings where access to continuing education and specialised training for pediatric care is minimal [13, 21].

In Ghana, medication administration errors are further complicated by systemic barriers that plague the healthcare system, particularly in rural areas. Yendi Municipal Hospital, the setting for this study, serves as a critical healthcare provider in Northern Ghana, catering to a large rural population. Rural hospitals in Ghana are often understaffed and under-resourced, leading to an overreliance on nurses who may not have the specialised training needed for pediatric care. Pediatric nurses in these settings often face a dual challenge: they are tasked with administering complex, weight-based doses for children, managing high patient loads and working in environments with insufficient resources [22]. These factors combine to create a setting where the risk of MAEs is particularly high yet remains understudied.

Previous research on healthcare delivery in Ghana has revealed that rural hospitals face substantial barriers to ensuring safe medication practices. In many cases, nurses in rural settings lack access to continuing education programs that could provide updates on the latest pediatric medication safety protocols [16]. Additionally, many rural healthcare workers rely on adult-dosing guidelines, even when treating pediatric patients, due to the lack of pediatric-specific medications and clear protocols [18]. These issues are exacerbated by the high patient-to-nurse ratios in rural hospitals, where nurses may manage up to three times the number of patients as their counterparts in urban hospitals [19, 23, 24]. This situation puts the nurses and their patients at a heightened risk of medication errors, particularly in pediatric care, where precise dosing is critical.

Given the unique challenges pediatric nurses face in rural Ghana, there is an urgent need for targeted interventions that address the specific factors contributing to MAEs in this context. By focusing on Yendi Municipal Hospital, this study aims to provide a detailed examination of how nurses’ knowledge, practices, and working conditions affect the incidence of medication errors in pediatric care. The study explored the key contributing factors to MAEs, including inadequate pediatric training, reliance on adult medication guidelines, high workloads, and lack of access to critical resources. Additionally, this paper assessed how cultural factors, such as stigma surrounding healthcare and fear of punishment for errors, contribute to the underreporting of medication errors, further complicating efforts to address the issue [21].

By investigating these factors in a rural, low-resource setting, this study seeks to fill a crucial gap in the existing literature on MAEs, which has largely focused on adult populations or urban healthcare environments. The findings from this study will not only provide evidence-based recommendations for improving pediatric care at Yendi Municipal Hospital. Still, they will also offer insights that can be applied to similar rural settings across Sub-Saharan Africa. These recommendations will focus on enhancing pediatric nurse training, improving medication safety protocols, and creating supportive reporting mechanisms that encourage nurses to report errors without fear of punishment [23, 24].

The current study seeks to fill a critical gap in the literature by focusing on the specific factors contributing to MAEs in pediatric care within a rural Ghanaian context. By thoroughly examining the knowledge, practices, and challenges nurses face at Yendi Municipal Hospital, the study will provide targeted recommendations to reduce medication errors and improve patient safety By addressing these issues in a rural, resource-limited setting. The findings will offer valuable insights into how similar healthcare systems across Sub-Saharan Africa can improve the quality of pediatric care through better medication management and error-reporting protocols.

## Methods and materials

### Research design

This study employed an institution-based descriptive cross-sectional survey to investigate pediatric nurses’ perceptions of factors contributing to medication administration errors (MAEs) at Yendi Municipal Hospital. This design was chosen because it allows for the simultaneous collection of data on nurses’ attitudes and perceptions at a single point in time, enabling the identification of prevalent factors influencing MAEs. This study uses a cross-sectional design to gather insights to inform targeted interventions to improve medication safety practices in pediatric care.

### Research setting

The study was conducted at Yendi Municipal Hospital, a key healthcare facility in the Yendi Municipality of Northern Ghana. Established in 1947, the hospital has a total capacity of 170 beds, with 30 beds specifically allocated to the pediatric unit. The pediatric unit admits approximately 8 to 10 patients weekly, resulting in more than 100 admissions annually. The common conditions treated in the hospital include: respiratory infections, malnutrition, and malaria, which are common pediatric illnesses in this rural region.

Yendi Municipal Hospital serves an estimated population of 2,500 individuals from nearby districts, most of which are rural. The hospital employs 222 staff members, including nurses, doctors, and administrative personnel. However, this study specifically focused on nurses involved in pediatric care, including registered nurses, nursing officers, and enrolled nurses who handle direct patient care. Yendi Municipal Hospital was selected as the research site due to its central role in pediatric care, providing a relevant context for understanding the factors contributing to MAEs. Its relatively high patient volume and representation of rural healthcare challenges make it an ideal setting for exploring pediatric nurses’ perceptions regarding MAEs.

### Study population

The study population consisted of Registered nurses and Licensed Practice Nurses directly involved in pediatric care at Yendi Municipal Hospital. These nurses were categorised based on their educational qualifications and years of experience, as follows:

1. Certificate Nurses: Nurses who complete a two-year training program are responsible for basic nursing duties, such as administering medications and monitoring pediatric patients under supervision.
2. Diploma Nurses: Nurses who completed a three-year training program are responsible for more advanced tasks, including pediatric unit management, overseeing junior staff, and ensuring safe medication administration.
3. Degree Nurses: Nurses with a bachelor’s degree (four-year program) are involved in complex decision-making regarding pediatric care, including managing critical cases, calculating medication doses, and developing treatment plans.

In addition to these main categories, the study also included:

- Ancillary Nurses: Nurses with basic training who assist in general patient care but are not involved in complex decision-making for pediatric patients.
- Professional Nurses: Fully licensed nurses who provide independent care and lead pediatric clinical interventions.
- National Service Nurses: Recent graduates fulfilling their mandatory one-year national service, assisting in pediatric care under supervision.

All participating nurses were registered with the Ghana Nursing Council, ensuring they met national standards for professional nursing practice. A significant portion of the study population had also received pediatric-specific training, which provided them with specialised skills in pediatric medication administration. This range of educational backgrounds and experience levels offered a comprehensive view of the factors contributing to MAEs in pediatric care. This aligns with the study’s investigation of nurses’ perceptions of MAEs.

### Inclusion/exclusion criteria

Eligible nurses were identified in collaboration with the hospital administration. A random selection process was used within each pediatric unit, employing “yes” or “no” slips to ensure an unbiased selection of participants from different departments.

Inclusion Criteria:

- Registered nurses and Licences Practice nurses with at least a, certificate, diploma, or degree.
- The nurses should have at least one year of experience in pediatric care.
- They should be willing to participate in the study.

Exclusion Criteria:

- Nurses or Licences Practice nurses on annual or maternity leave during the data collection period.
- Nurses who are unwell or attending off-site training.
- Nurses who are not directly involved in pediatric care, such as those mainly in administrative roles.

This selection process ensured the study population was directly involved in pediatric medication administration. This allowed for a focused investigation of the perceived factors contributing to MAEs among the nursing staff.

### Sampling method and calculation

The study employed a combination of purposive and stratified random sampling to ensure a representative sample of pediatric nurses.

- Purposive sampling was used to select nurses specifically involved in pediatric care, ensuring their relevance to the study’s aim of investigating MAEs.
- Stratified random sampling ensured proportional representation across different nursing categories (certificate, diploma, degree) and pediatric units (neonatal, pediatric wards, outpatient departments).

### Sample size calculation

The sampling method combined purposive sampling to select pediatric care nurses and stratified random sampling to ensure representation across different units and educational levels. The sample size was calculated using Yamane’s formula [25], which is appropriate for large population studies. The sample size for the study was determined as:

$$\:n=\frac{N}{{1+N\left(e\right)}^{2}}$$

Where:

- n is the desired sample size,
- N is the total population (222),
- e is the margin of error (0.05).

$$\:n=\frac{222}{{1+222\left(0.05\right)}^{2}}$$

So, *n*=143.

The calculated sample size was 143 nurses.

#### Proportionate sampling

The total population was divided proportionately among different nurse categories:

- Certificate Nurses: 53.8% (77 nurses).
- Diploma Nurses: 30.8% (44 nurses).
- Degree Nurses: 11.8% (17 nurses).
- Ancillary Nurses: 3.5% (5 nurses).

Nurses were then randomly selected within each subgroup using the lottery method to ensure an unbiased sample, enabling a comprehensive investigation of pediatric nurses’ perceptions of factors contributing to MAEs.

### Development and validation of the questionnaire

The questionnaire was developed following a comprehensive literature review and in consultation with pediatric nursing experts. Several steps were taken to ensure its validity and reliability:

1. Face validity: A panel of experts reviewed the questionnaire for clarity, relevance, and comprehensiveness.
2. Pre-test: A small sample of nurses not included in the main study participated in a pre-test to refine the questions and ensure they were understandable and relevant.
3. Factor analysis: Factor analysis was performed to confirm the underlying structure of the questionnaire items.
4. Reliability testing: Cronbach’s alpha was used to assess the internal consistency of the questionnaire, yielding a reliability score of 0.85, indicating high reliability.

These steps ensured the questionnaire was valid and reliable, making it an effective tool for exploring pediatric nurses’ perceptions of factors contributing to MAEs. Find the questionnaire used for data collection in; Supplementary File 1.

### Data collection procedures

Data collection occurred at Yendi Municipal Hospital from January to February 2022. Trained research assistants approached potential participants during working hours, provided study information, and obtained informed consent. In the Northern region of Ghana, where there is a higher proportion of male nurses, this gender distribution was reflected in the study sample. The stratified sampling technique ensured that the questionnaire was administered across various units, with each participant taking 20–25 min to complete it.

### Data analysis

Data were coded and analysed using SPSS version 26.0 and Microsoft Excel 2016. Descriptive statistics summarised demographic details and nurses’ perceptions about factors contributing to MAEs. Bivariate analysis using chi-square tests examined relationships between socio-demographic characteristics and MAEs, with a p-value < 0.05 considered statistically significant.

### Data management

Data management is vital for analysing pediatric nurses’ perceptions of medication administration errors (MAEs) at Yendi Municipal Hospital. Structured questionnaires collected data while ensuring participant anonymity. Paper records were stored in locked cabinets, and digital data was kept in password-protected databases. Confidentiality was upheld by removing personal identifiers and restricting access to authorised personnel. Double-entry procedures and audits were used to maintain data integrity. After analysis, paper records were shredded, and digital data were securely stored for five years before deletion. This comprehensive approach ensures reliable findings and informs future pediatric care policies.

### Ethics

The Ghana Health Service ethics committee approved the research protocol with the document reference number GHS-ERC069/03/22. Written informed consent was obtained from all participants. Participation was voluntary, and confidentiality was maintained throughout the study. Participants were given the option to withdraw from the study at any time.

### Justification for the study

The study aimed to investigate pediatric nurses’ perceptions of factors contributing to MAEs at Yendi Municipal Hospital, Ghana. This setting was chosen because it represents the challenges of rural healthcare, making the findings applicable to similar settings both locally and globally. The study fills a gap in research on MAEs in pediatric care, particularly in rural areas, where nurses face unique challenges related to resource limitations, high patient-to-nurse ratios, and lack of continuous training.

## Results

The study recruited 143 nurses, with a mean age of 29.7 ± 3.8 years. The majority of the participants (66.4%) were younger than 30 years, and males constituted a slightly higher proportion (53.9%) compared to females (46.1%). The religious distribution showed 81.1% of participants were Muslim, while the remaining 18.9% were Christian. A majority 71.3% of the respondents were married, and more than half 53.8% held a certificate in nursing. Additionally, 49.6% of the nurses had between 1 and 5 years of working experience, 51.8% worked in mixed wards, and 48.2% worked in pediatric wards. Table 1, as shown below, shows the Socio-demographic characteristics of the respondents.

**Table 1:** Socio-demographic characteristics of respondents

Variable		Frequency
**Age (years)**	mean, SD	29.7, 3.8
**Age-group**		
	< 30	95 (66.4)
	30++	48 (33.6)
**Sex**	Male	77 (53.9)
	Female	66 (46.1)
**Religion**	Christianity	27 (18.9)
	Islam	116 (81.1)
**Marital Status**	Single	41 (28.7)
	Married	102 (71.3)
**Educational level**	Certificate	77 (53.8)
	Diploma	44 (30.8)
	1st Degree	17 (11.8)
	Others	5 (3.5)
**Years of working**	< 1	39 (27.3)
	1–5	71 (49.6)
	6–10	27 (18.9)
	> 10	6 (4.2)
**Type of unit/ward**	Only paediatrics	69 (48.2)
	Mixed ward	74 (51.8)

**Table 2:** Perceptions about factors contributing to MAEs among nurses (*n* = 143)

Perception	Agree (%)	Neutral (%)	Disagree (%)
**Knowledge and Work Experience**			
Inadequate in-service training	91.6	-	8.4
Inadequate understanding of medical abbreviations	88.8	-	11.2
Inadequate supervision of new staff	92.3	-	7.7
Inadequate knowledge of MAEs	41.3	21.7	37.1
Work experience	83.2	-	16.8
Education level	60.8	-	39.2
**Work Environment and Attitudinal**			
Eagerness to sign out shifts	70.6	-	29.4
Improper handing-over process	88.8	-	11.2
Inadequate nursing staff	77.6	-	22.4
Poor working environment (e.g., lights)	81.1	-	18.9
**Documentation and Communication**			
Miscalculations of dosages	83.9	-	16.1
Illegible handwriting on medications	81.8	-	18.2
Errors in dosages/medication documentation	88.1	-	11.9
Poor nurse communication	86.7	-	13.3
Errors in medicine administration time	83.2	-	16.8
Using abbreviations	62.9	9.1	27.9
**Workload**,** Stress**,** and Distraction-Related Perceptions**			
Too much workload	82.5	0	17.5
Demanding attention from other patients	62.9	0	37.1
Phone call distractions	52.4	0	47.6
Interruption during drug administration	81.1	0	18.9
Long working hours	76.9	0	23.1
Feeling sleepy on duty	74.8	0	25.2
Nurses’ psychological state	86.7	0	13.3
Forgetfulness among nurses	90.9	0	9.1

As seen in Table 2, Perceptions About Contributing Factors to MAEs, the nurses’ perceptions of factors contributing to MAEs were grouped into categories: knowledge and work experience, work environment and attitudes, documentation and communication, and workload, stress, and distractions.

- Knowledge and Work Experience: The majority (92.3%) believed that inadequate supervision of new staff contributed to MAEs, and 88.8% attributed errors to the inadequate understanding of medical abbreviations.
- Work Environment and Attitudinal Factors: A large proportion (88.8%) agreed that improper handing-over processes were a factor, and 81.1% felt that poor working environments (e.g., lighting) contributed to errors.
- Documentation and Communication: Illegible handwriting and errors in dosage documentation were considered significant contributors by 81.8% and 88.1% of respondents, respectively.
- Workload, Stress, and Distractions: The majority (82.5%) agreed that excessive workload contributed to MAEs, and 81.1% highlighted interruptions during drug administration as a contributing factor.

**Table 3:** Challenges that prevent MAE Reporting

Challenge	Agree (%)	Neutral (%)	Disagree (%)
Fear of rebuke from physicians	72.7	0	27.3
Unclear definition of MAE reporting	82.5	0	17.5
Unawareness of MAEs	66.4	0	33.6
Less attention is given to MAEs	70.6	0	29.4
Nurses’ fear of losing their license	74.8	0	25.2
Negative feedback for reporting MAEs	82.5	0	17.5
Fear of adverse consequences	76.2	0	23.8

Table 3 above, Several barriers to reporting MAEs were identified. Most notably, 82.5% of nurses cited unclear definitions of an MAE as a significant barrier, while 74.8% feared losing their licenses. These findings highlight the need for clearer guidelines and a supportive reporting culture.

### Association between MAE and socio-demographic characteristics

The bivariate analysis of the socio-demographic characteristics reveals significant associations between certain factors and medication administration errors (MAEs) among the participants. Notably, the type of unit/ward showed a significant association with MAEs (X2 = 6.25, *p* = 0.012). The educational level also demonstrated a significant association, with certificate nurses having higher incidences of MAEs than those with diploma or degree qualifications (Fisher Exact test = 4.20, *p* = 0.036).

While not statistically significant, the analysis indicated that male nurses had a higher incidence of MAEs than their female counterparts. Furthermore, nurses with over ten years of working experience reported more MAEs than those without experience. These observations are detailed in Table 4.

**Table 4:** Association between MAE and socio-demographic characteristics

Variables	Response category	MAE		Test Statistics
		Yes	No	
Age of respondents	< 30 years	36 (37.9)	59 (62.1)	X^2^ = 0.83, *p* = 0.231
	> 30 years	22(45.8)	26(54.2)	
Sex	Male	34 (44.2)	43(55.8)	X^2^ = 0.89, *p* = 0.219
	Female	24(36.4)	42(63.6)	
Marital status	Single	17 (42.5)	23 (57.5)	X^2^ = 0.750, *p* = 0.911
	Married	41 (40.2)	62(59.8)	
Religion	Christianity	14 (51.9)	13 (48.1)	X^2^ = 1.76, *p* = 0.134
	Islam	44 (37.9)	72 (62.1)	
Educational level	Certificate	35 (45.5)	42 (54.5)	Fisher Exact test = 4.20, *p* = 0.036
	Diploma	17(38.6)	27 (61.4)	
	1st degree	6(35.3)	11(64.7)	
	Others	0 (0.0)	5 (100)	
Years of working	< 1	17 (43.6)	22(56.2)	Fisher Exact test = 1.06, *p* = 0.806
	1–5	29 (40.8)	42 (59.2)	
	6–10	9 (33.3)	18 (66.7)	
	> 10	3 (50.0)	3(50.0)	
Type of unit/ward	Only pediatric	26 (54.2)	22 (45.8)	X^2^ = 6.25, *p* = 0.012
	Mixed ward	32(33.7)	63 (66.3)	

## Discussions

The research findings provide critical insights into the factors contributing to medication administration errors (MAEs) among nurses at Yendi Municipal Hospital, a rural healthcare facility in Northern Ghana. As frontline healthcare providers, nurses play an indispensable role in ensuring safety and proactive patient care. The study highlights their pivotal involvement in preventing MAEs within healthcare systems, particularly in resource-limited settings [26, 27].

This aligns with global movements toward achieving Sustainable Development Goals (SDGs), particularly SDG 3 (Good Health and Well-Being), SDG 4 (Quality Education), and SDG 5 (Gender Equality), which emphasise universal healthcare, educational advancement, and reducing healthcare disparities [25, 28–30].

### Contributors to MAEs

Multiple systemic and individual factors were identified as contributors to MAEs. Among the most significant were inadequate in-service training, lack of understanding of medical abbreviations, and insufficient supervision of new staff. These findings are consistent with a wealth of literature pointing to the need for continuous education and effective supervision to reduce the risk of medication errors [31, 32]. For example, Wright (2013) underscores the critical importance of ongoing training and mentorship in improving medication safety [20]. A systematic review by Kuitunen et al. (2021) also found that inadequate knowledge of pharmacology and poor communication were key risk factors for MAEs in hospital settings [33]. However, some studies, such as by Chua et al. (2017), suggest that education alone may not address the systemic issues underlying MAEs [34]. The author argue for a broader, systems-based approach incorporating organisational change and individual training.

Pediatric care presents additional challenges due to the complexity of dosage adjustments necessitated by children’s rapid growth and the variation in metabolic rates. Age-related pharmacokinetics and pharmacodynamics are critical in pediatric medication management [33, 35]. As Ghaleb et al. (2006) highlighted, errors in pediatric dosing can have severe consequences due to the narrow therapeutic windows for many pediatric medications [24]. This study’s findings echo these concerns, emphasising that nurses must comprehensively understand pediatric pharmacology to administer medications safely. However, while research often emphasises training as a solution, Vaismoradi et al. (2020) caution that psychological barriers such as fear of blame and guilt may impede nurses from fully engaging with training and error-reporting processes [36].

The analysis also uncovered systemic factors such as impatience to complete shifts, ineffective handover procedures, and poor working conditions, including staff shortages and excessive workloads, as significant contributors to MAEs. Documentation challenges, including illegible handwriting, dosage miscalculations, and poor communication, further exacerbated the risk of errors. The findings align with a study by Armitage and Knapman (2003), which emphasised that systemic factors, such as workload and staffing issues, are critical contributors to medication errors [37]. However, the literature on solutions is mixed. While some studies, such as Thomas (2019), support the introduction of electronic health records (EHRs) to address documentation issues [17], others, like Institute of Medicine. (2006), argue that poorly designed EHR systems can introduce new types of errors [30], suggesting that technological solutions must be carefully managed to avoid unintended consequences.

### Significant associations

The bivariate analysis revealed that nurses working in mixed wards reported higher incidences of MAEs than those in pediatric-only wards, suggesting that specialisation within pediatric units may help reduce errors. This finding is consistent with research by Holden et al. (2011), which found that unit specialisation is associated with lower error rates, as specialised wards typically foster more focused and consistent practices [38]. In mixed wards, nurses are exposed to a broader range of patient needs and conditions, which may increase the likelihood of medication errors due to the complexity and variability of care required (24–27, 42-). These findings underscore the importance of providing targeted support and training to nurses in mixed wards. However, contrasting findings were reported by Tang et al. (2007), who found no significant difference in error rates between specialised and mixed wards, suggesting that factors beyond specialisation, such as the quality of teamwork and communication, may play a more significant role in determining error rates [39].

Educational level was another significant factor associated with MAEs, with certified nurses exhibiting higher error rates than those with diploma or degree qualifications. This result supports the findings of Tang et al. (2007), who demonstrated that advanced education in nursing correlates with improved medication safety and reduced error rates [39]. Higher educational attainment typically involves more comprehensive training in pharmacology, patient safety, and error prevention strategies, contributing to reducing MAEs. However, some studies, such as Carayon et al. (2014), challenge this notion, suggesting that experience, rather than education level, plays a more critical role in preventing errors [40]. This suggests ongoing education and mentorship should complement formal qualifications to achieve the best outcomes.

Interestingly, while not statistically significant, a trend indicated that male nurses and those with over ten years of experience reported more MAEs than their female and less experienced counterparts. Gender differences in error reporting have been explored in studies such as Leape et al. (1995), though findings remain inconclusive [41]. While some researchers attribute higher error rates among male nurses to differences in communication styles or attitudes toward risk, others suggest that these discrepancies may be context-dependent and require further investigation. Similarly, the relationship between experience and error rates is complex. At the same time, more experienced nurses may face greater demands and responsibilities, leading to higher reported errors. They may also be more likely to report errors due to a stronger sense of professional accountability (23, 40, 41).

### Barriers to reporting

Fear of reprimand, ambiguous error-reporting protocols, and lack of awareness were prominent barriers to reporting MAEs. These findings align with existing literature on nurses’ psychological barriers in reporting errors. A study by Armitage and Knapman, (2023) found that fear of blame and negative appraisal often prevent nurses from reporting errors, particularly in punitive work environments [37]. The psychological toll of guilt also plays a role, as highlighted by Tank (2007), who argues that emotional factors must be considered when designing interventions to promote error reporting [39]. Establishing a non-punitive environment that encourages open reporting is critical for improving patient safety. This is consistent with recommendations from the World Health Organization (WHO, 2009), which advocate for fostering a safety culture in healthcare settings [29] .

### Proposed solutions

To reduce MAEs, a comprehensive and multifaceted approach is needed. This study recommends regular in-service training to address knowledge gaps, especially in medical abbreviations and pediatric dosing. Strengthened supervision, particularly for new staff, can help ensure adherence to best practices and reduce the risk of errors. Improved handover procedures are also essential, particularly in mixed wards with higher error rates. Additionally, establishing clear, non-punitive reporting protocols is crucial to encouraging error reporting and reducing the fear of reprimand [38, 42].

Organisational support is also key. Reducing workload and providing adequate staffing are necessary steps to minimise stress and fatigue among nurses, which are significant contributors to errors. Enhanced communication within nursing teams and better documentation protocols can further improve patient safety. Studies by Carayon et al. (2014) advocate for such comprehensive strategies to reduce errors in healthcare [40].

### Limitations of the study

This study was conducted exclusively at Yendi Municipal Hospital, which limits the generalizability of the findings to other healthcare settings, particularly non-rural hospitals. The reliance on self-reported data from nurses introduces the potential for response bias, which could affect the accuracy of the results. The cross-sectional design also provides a snapshot of MAEs at one point in time, without accounting for changes over time. Furthermore, the focus on pediatric nurses may limit the applicability of the findings to other specialities, and perspectives from other healthcare professionals, such as doctors and pharmacists, were not included.

### Significance of the study

The study provides valuable insights into the factors contributing to MAEs in pediatric care at Yendi Municipal Hospital. It highlights the need for continuous education, particularly in understanding medical abbreviations and proper medication administration practices. The study also emphasises the importance of fostering a non-punitive culture and establishing clear reporting protocols to encourage the accurate reporting of errors. A comprehensive approach involving policy revisions, enhanced communication, better organisational support, and ongoing training is recommended to improve medication safety and care quality for pediatric patients.

### Recommendations

Based on the results from the study, It is recommended that there is the need for ward-in -charges, Ministry of Health and other policymakers to:

1. Implement regular in-service training to address knowledge gaps, particularly in medical abbreviations and medication practices.
2. Strengthen supervision for new staff to ensure adherence to best practices and reduce errors.
3. Develop tailored strategies, including focused training and additional support for nurses with higher error rates in mixed wards.
4. Establish clear, non-punitive reporting protocols to encourage the reporting of MAEs and reduce the fear of reprimand.
5. Encourage nurses to pursue higher qualifications, as advanced education correlates with fewer errors.
6. Improve working conditions to reduce workload and stress, thus minimising errors.

## Conclusion

This study underscores the complexity of MAEs and the systemic factors contributing to these errors among nurses in rural healthcare settings. A concerted effort is required to enhance training, improve communication, and strengthen organisational support to address the root causes of MAEs. By targeting these areas and implementing specific interventions, healthcare systems can significantly improve medication safety and the overall quality of care for patients.

## Electronic supplementary material

Below is the link to the electronic supplementary material.

Supplementary Material 1

## Data Availability

On reasonable request, the corresponding author will make all datasets used and analysed during the present study accessible to the reader.

## Abbreviations

ADEs: Adverse drug events
MAE: Medication administration errors
