# Supplementary material for: Investigating pediatric nurses’ perceptions of factors contributing to MAEs at Yendi hospital, Ghana
Source: BMC Pediatr. 2024 Dec 3;24:792. doi: 10.1186/s12887-024-05269-x (PMC11613571; doi:10.1186/s12887-024-05269-x)
Supplement: Supplementary file 1 — Supplementary Material 1 [file 12887_2024_5269_MOESM1_ESM.docx]

**Supplementary File 1**

**Data Collection Tools: Questionnaire**

**Informed consent**

I am a resident of the Ghana College of Nurses and Midwives and conducting a study on predictors of medication administration error among nurses caring for children in the Yendi municipality hospital. Your responses will be treated confidentially and would be used for academic purposes only. You can choose to answer all or some of the questions without any penalty imposed on you. The information you will provide will be kept confidential and used only for academic purposes. Your participation in the study will be greatly appreciated.

**Section A: Biodata of respondents**

1. How old are you? …………………………………..
2. Indicate your sex a. male ( ) b. female ( )
3. Indicate your marital status a. single ( ) b. married ( ) c. separated ( ) d. others (specify)……………………………………………….
4. Indicate your highest educational qualification a. certificate ( ) b. diploma ( ) c. degree ( ) d. others (specify)…………………………………………………..
5. How long have you practiced as a nurse? a. < 1year ( ) b. 1 – 5 years ( ) c. 6– 10 years ( ) d. < 10 year ( )
6. Indicate your unit (specify)…………………………………………………..

**Section B: Factors contributing to medication administration errors among nurses**

1. Do you consider the following as factors contributing to medication administration error among nurses in this hospital?

| **Item** | **Agree** | **Neutral** | **Disagree** |
| --- | --- | --- | --- |
| Education |  |  |  |
| Work experience |  |  |  |
| Inadequate number of nurses in each working shift |  |  |  |
| Nurses on this unit have limited knowledge about medications |  |  |  |
| Pharmacy delivers incorrect doses to this unit |  |  |  |
| Abbreviations are used instead of writing the orders out completely |  |  |  |
| The packaging of many medications is similar |  |  |  |
| Physicians change orders frequently |  |  |  |
| Different medications look alike |  |  |  |
| Physicians’ medication orders are not legible |  |  |  |

1. Do you view these as factors also contributing to MAE?

| Item | **Agree** | **Neutral** | **Disagree** |
| --- | --- | --- | --- |
| Administration Time Error |  |  |  |
| Not arranging folders based on bed number |  |  |  |
| Trolley not well equipped |  |  |  |
| Serving medication of one patient to another patient  Administering drug at wrong time |  |  |  |
| Administering drug over ordered duration |  |  |  |
| Administering drug not prescribed |  |  |  |
| Diluting drug not supposed to be diluted |  |  |  |
| Drug Documentation Error |  |  |  |
| Forgetfulness |  |  |  |
| Psychological state of the nurse |  |  |  |
| Nurse feeling sleepy on night duty |  |  |  |
| Poor working conditions |  |  |  |
| Poor staffing on the ward |  |  |  |
| Long consecutive hours of working |  |  |  |
| Poor communication with other  Nurses |  |  |  |
| No documentation of medication  administration procedure |  |  |  |
| Illegible handwriting of  medication orders |  |  |  |
| Use of abbreviations |  |  |  |
| Interruption during drug  Administration |  |  |  |
| Receiving telephone phone calls from other units |  |  |  |
| Demanding of attention by other Patients |  |  |  |
| Poor supervision of new staff or student nurses |  |  |  |
| Others |  |  |  |

1. In own opinion, during which shift does drug administration error occurs most? (a) Morning shift [ ] (b) Afternoon shift [ ] (c) Night shift [ ]

**Section C: Challenges of preventing medication administration errors among nurses**

1. Do you consider the following as challenges of preventing medication administration errors among nurses in this hospital?

| **Item** | **Agree** | **Neutral** | **Disagree** |
| --- | --- | --- | --- |
| Fear of adverse consequences |  |  |  |
| A negative response from reporting MAEs |  |  |  |
| Nurses fear losing their license |  |  |  |
| No harm to patient following drug administration error |  |  |  |
| Less emphasis is placed on medication errors |  |  |  |
| Nurse are not aware that an error occurred |  |  |  |
| Lack of clear definition for MAE |  |  |  |
| Nurses fear rebuke from physician |  |  |  |
| Others (specify) |  |  |  |

**Section D: Ways of reducing medication administration errors among nurses**

1. Do you consider the following as ways of reducing medication administration errors among nurses in this hospital?

| **Item** | **Agree** | **Neutral** | **Disagree** |
| --- | --- | --- | --- |
| Improved monitoring of medication administration |  |  |  |
| Inservice training for nurses on MAEs |  |  |  |
| Protecting nurses who report MAEs to management |  |  |  |
| Banning the use of phones in the ward |  |  |  |
| Employing a second person to assess mediations |  |  |  |
| Publishing nurses who commit MAEs |  |  |  |
| A positive response from reporting MAEs |  |  |  |
| A clear definition for MAE |  |  |  |
| Others (specify) |  |  |  |

Thank you for the time.
